# Serum C-reactive protein levels are associated with clinical pregnancy rate after *in vitro* fertilization among normal-weight women

**DOI:** 10.3389/fendo.2023.934766

**Published:** 2023-01-19

**Authors:** Huixia Zhang, Xin Li, Fan Zhang, Fei Li, Haixia Jin, Yingchun Su, Gang Li

**Affiliations:** ^1^ Centre for Reproductive Medicine, The First Affiliated Hospital of Zhengzhou University, Zhengzhou, China; ^2^ Henan Key Laboratory of Reproduction and Genetics, The First Affiliated Hospital of Zhengzhou University, Zhengzhou, China

**Keywords:** C-reactive protein, inflammation, body mass index, clinical pregnancy, live birth, *in vitro* fertilization

## Abstract

**Objective:**

To assess whether low-grade inflammation, measured by serum high-sensitivity C-reactive protein (hsCRP) levels, is associated with *in vitro* fertilization (IVF) outcomes.

**Design:**

A retrospective study.

**Setting:**

University-affiliated IVF center.

**Patient(s):**

In the present study, 875 women of normal weight who underwent their first fresh embryo transfer (ET) cycles for IVF treatment were divided into three groups according to serum concentrations of hsCRP.

**Intervention(s):**

Serum from women undergoing IVF was collected on days 2-4 of a spontaneous menstrual cycle prior to the commencement of ovarian stimulation.

**Main Outcome Measure(s):**

The IVF outcomes included implantation, biochemical pregnancy, clinical pregnancy, miscarriage and live birth rates.

**Result(s):**

The women were divided into three groups according to the baseline serum levels of hsCRP as follows: low hsCRP (<1 mg/L; n=517), medium hsCRP (1-3 mg/L; n= 270), high hsCRP (>3 mg/L; n=88). The maternal age was similar among the three groups. The women in the high and medium hsCRP group had significantly higher BMI compared with those in the low hsCRP group. The protocol of controlled ovarian hyperstimulation, the gonadotropin dose administered, the serum estradiol levels, progesterone levels and the endometrial thickness on the day of triggering, as well as the number of retrieved oocytes, fertilized oocytes and good quality embryos, and the oocyte maturation rate were similar among the three groups. Implantation, biochemical pregnancy and clinical miscarriage rates did not differ significantly were not significantly different among three groups. The clinical pregnancy rate was significantly lower in the high hsCRP group compared with that in the low hsCRP group (50.0% versus 63.4%; P<0.0167), which contributed to a significant decrease in birth rate (39.8% versus 53.8%; P<0.0167). High serum hsCRP levels was found to be a factor affecting live birth rate

**Conclusion(s):**

Among women of normal weight undergoing their first IVF treatment, it was found that low-grade inflammation was associated with reduced clinical pregnancy and live birth rates following fresh ET cycles.

## Introduction

Inflammatory molecules and immune cells play an essential role in the functional processes of ovarian folliculogenesis, ovulation and embryo implantation (1). The increased expression of the pro-inflammatory cytokines, tumor necrosis factor (TNF-α), interleukin (IL)-6 and IL-8, has been shown to be associated with ovulatory dysfunction and impaired oocyte quality (2, 3). The genomic and transcriptomic analyses on follicular cells and endometrial fluid from women who achieved pregnancy and those who did not following *in vitro* fertilization (IVF) has revealed that an increased local inflammatory state was a key factor limiting IVF success (4, 5). Moreover, circulating levels of pro-inflammatory molecules, such as resistin, leptin, and IL-22 have been found to be elevated in patients with recurrent miscarriage and repeated implantation failure (6). These findings demonstrate that a disrupted balance of pro- and anti-inflammatory factors may underlie reproductive failure.

High-sensitivity C-reactive protein (hsCRP) is a well-established marker of systemic inflammation in clinical practice, and is a liver-derived acute phase protein produced in response to the release of IL-6 from activated immune cells, such as macrophages and adipocytes (7). In response to acute infection and tissue damage, serum levels of CRP increase rapidly by ~1,000-fold within several hours, whereas moderately elevated levels of hsCRP are considered to reflect chronic low-grade inflammation that is caused by various factors (7–11). Increased serum CRP levels have been linked to reduced fecundability and poor ART outcomes (11, 12). In the Effects of Aspirin in Gestation and Reproduction (EAGER) trial, among women with higher levels of systemic inflammation (hsCRP≥1.95 mg/L) attempting conception, the daily use of low-dose aspirin(LDA) improved clinical pregnancy and live birth rates compared with women assigned the placebo. Such a significant effect of LDA was identified in women with a body mass index (BMI) <25 kg/m^2^, although no statistically significant effect was found in overweight/obese women (13). It has been suggested that BMI is strongly and positively associated with serum CRP concentrations (14–16). These findings indicate that BMI is a confounder of the association between low-grade inflammation and fertility.

However, the association between serum CRP concentrations measured measured prior to ovarian stimulation and IVF success has not yet been fully investigated. Buyuk et al. (14) showed a significant decrease of CRP levels in pregnant women compared with non-pregnant women, whereas others found similar CRP levels in both groups (17, 18). These studies had a small sample size, and none of them assessed the association between baseline CRP concentrations and live birth rates following IVF. To date, to the best of our knowledge, there are no available data on the effects of elevated hsCRP levels on IVF outcomes in women of normal weight.

The aim of the present study was to examine whether low-grade inflammation, measured by serum hsCRP levels prior to ovarian stimulation, is associated with IVF outcomes independently from adiposity, using a larger sample of women. In addition, the present study assessed whether the baseline serum CRP levels were independently associated with the possibility of live birth. Our study defined low-grade inflammation as serum hsCRP levels>3 mg/L. This cut-point has been chosen based on the Center for Disease Control and Prevention and the American Heart Association recommendations, which defined hsCRP levels of >3 mg/L as high (19). Clear CRP thresholds are considered to denote inflammation levels (<1 = ‘low’, 1-3 = ‘medium’, >3mg/L= ‘high’) in studies on cardiovascular disease, depression and cancer (19–21). Therefore, we carried out analyses using the above cut-off values as indicators of hsCRP levels.

## Materials and methods

### Study population and design

We retrospectively reviewed women who underwent their first fresh ET cycles for IVF/ICSI treatment in the reproductive medical center of the First Affiliated Hospital of Zhengzhou University between 1 July 2021 and 31 November 2021. In the course of routine workup for treatment, women underwent transvaginal ultrasound, testing of baseline sex hormones serum levels on day 2-4 of menstruation. As part of their routine workup, women at our center also underwent CRP assessments. In total, 875 normal-weight women (BMI 18.5-25kg/m^2^) who had their serum hsCRP measured prior to initiation of ovarian stimulation were included. Baseline serum concentrations of hsCRP were quantified by immunoturbidimetric assay using Aristo autoanalyzer. Exclusion criteria were: 1) women who had all their oocytes or embryos electively cryopreserved; 2) cycles that were cancelled because of no transferable embryos or personal reasons;3) women with hsCRP≥10 mg/L, a level which usually indicates the presence of acute infection or injury as opposed to chronic low-grade inflammation; 4) women with autoimmune disorders, such as antiphospholipid syndrome, sjogren’s syndrome and systemic lupus erythematosus, etc. Data from oocyte donation cycles were excluded. To investigate the relation of low-grade inflammation to IVF outcomes, three groups were established based on serum hsCRP levels (low, <1 mg/L; medium, 1-3 mg/L; high, >3 mg/L). Finally, implantation rates, clinical pregnancy rates, live birth rates and miscarriage rates were compared between the groups, we then used binary logistic regression model to examine the effect of hsCRP levels on the probability of live birth. The study was a retrospective study approved by the Medical Ethics Committee, and written informed consent was obtained from all women during the first consultation.

The data in current study were collected from the Clinical Reproductive Medicine Management System/Electronic Medical Record Cohort Database (CCRM/EMRCD) at our center. The baseline characteristics were the woman’s age, BMI, AMH, AFC, the baseline FSH, previous miscarriages, and the type and the duration of infertility, infertility factors (tubal factor, male factor, diminished ovarian reserve, polycystic ovary syndrome, endometriosis, and unexplained infertility). The cycle characteristics included the type of ART (IVF or ICSI), the stimulation protocol for fresh ET, the total gonadotropin dose, the number of retrieved, mature and fertilized oocytes, the number of good quality embryos, the number of frozen embryos, and the number of transferred embryos.

The implantation rate was calculated as the number of gestational sacs observed by vaginal ultrasound divided by the number of transferred embryos. Clinical pregnancy rate was calculated as the number of women with ultrasound detected presence of one or more gestational sacs after ET divided by the number of women undergoing ET.

Biochemical pregnancy was defined as a pregnancy diagnosed only by the testing for HCG in serum or urine and did not develop into a clinical pregnancy. The clinical miscarriage rate was calculated as the number of women with pregnancy loss before 20 weeks of gestation divided by the number of women with clinical pregnancy. Live birth was defined as delivery of any viable infant after 20 weeks of gestational age. Live birth rate was calculated by dividing the number of live births by the number of transfer cycles.

### Treatment protocol

Ovarian stimulation was performed with a starting dose of exogenous gonadotrophin (ranging from 112.5 to 300 IU) based on the patient’s age, BMI and ovarian function. The subsequent dose was continuously adjusted to meet patient’s needs. Follicle growth was monitored by transvaginal ultrasound and the serum levels of estradiol, progesterone and LH were measured regularly. When the dominant follicles larger than 16mm accounted for more than 2/3 of all follicles or a follicle reached 20mm in mean diameter, final follicular maturation and ovulation was triggered with the use of Aizer 250 μg (Merck Serono, Italy) or hCG 2,000 IU (Zhuhai Lizhu Medicine). Oocyte retrieval was performed under the guidance of transvaginal ultrasound 36-37 hours after hCG administration, followed by fresh embryo transfers on day 3 or day 5.

### Statistical analyses

Statistical analysis was performed using SPSS 26.0 software. Categorical variables were expressed as frequency (percentages) and continuous variables as means ± standard deviation. We used chi-square tests and Bonferroni correction to compare differences in categorical variables. Comparisons of continuous variables among independent groups were performed using Kruskal-Wallis test or one-way ANOVA, with a Bonferroni correction for multiple comparisons. We included some clinical variables as potential confounders that may have interacted with the effect of hsCRP on pregnancy outcomes. A multivariable logistic regression analysis was used to examine the effect of hsCRP categories on the probability of live birth. Adjusted odds ratios (aORs) with 95% confidence intervals (CIs) were estimated. P values were two-sided and a P value of <0.05 defined statistical significance (adjusted P-value following Bonferroni was 0.0167).

## Results

A total of 875 women undergoing their first fresh ET cycle for IVF treatment were included in this research. The median serum concentration of hsCRP was 0.8 mg/L, ranging from 0.01 to 9.6 mg/L, with overall wide range of hsCRP in study subjects. Of all women, 517 (59.1%) had low (<1 mg/L), 270 (30.9%) had medium (1-3 mg/L) and 88(10.0%)had high (>3 mg/L) levels of hsCRP. The basic characteristics of the three groups were shown in **Table 1**. The women’s median age was 33.8 years and ranged from 20 to 49 years, there was no statistical difference in age among the three groups. As expected, with increasing serum hsCRP levels, BMI increased, the medium and high hsCRP group had significantly higher BMI compared with the low hsCRP group (22.52 ± 1.70 versus 21.51 ± 1.77 kg/m^2^, 22.88 ± 1.43 versus 21.51 ± 1.77 kg/m^2^, respectively; P<0.001). There was a significant difference among three groups in baseline FSH levels (7.44 ± 3.05, 6.50 ± 2.22 and 6.51 ± 1.91mIU/ml, respectively; P<0.001). Groups were similar in terms of history of miscarriage, duration and the type of infertility. Apart from PCOS, other causes of infertility were similar among the different hsCRP groups, with tubal factor being the most common. In total, 18.2% of the women in the high hsCRP group were diagnosed with PCOS, which was significantly higher than that in the low hsCRP group (18.2% versus 7.4%; P<0.0167). This may lead to differences in the baseline FSH levels among groups.

**Table 1:** Basic clinical characteristics of women with low, moderate and high hsCRP.

	hsCRP (mg/L)			
Characteristic	<1 (n=517)	1-3 (n=270)	>3 (n=88)	*P* value
Age (years)	31.24 ± 4.79	31.81 ± 4.63	30.92 ± 4.79	0.173
BMI (kg/m^2^)	21.51 ± 1.77^a,b^	22.52 ± 1.70^a^	22.88 ± 1.43^b^	< 0.001
Baseline FSH (mIU/ml)	7.44 ± 3.05^a,b^	6.50 ± 2.22^a^	6.51 ± 1.91^b^	< 0.001
Baseline E_2_ (pg/ml)	44.14 ± 19.07	42.03 ± 16.20	45.87 ± 20.97	0.292
Baseline LH (mIU/ml)	5.34 ± 4.01	4.92 ± 3.37	5.56 ± 5.09	0.355
AMH (ng/ml)	3.40 ± 3.12	3.53 ± 2.88	3.97 ± 3.05	0.271
AFC	14.82 ± 61.76	12.74 ± 6.17	14.2 ± 6.03	0.844
History of miscarriage	213 (41.2%)	107 (39.6%)	30 (34.1%)	0.448
Infertility diagnosis				0.233
Primary infertility	250 (48.4%)	138 (51.1%)	51 (58.0%)	
Secondary infertility	267 (51.6%)	132 (48.9%)	37 (42.0%)	
Duration of infertility (years)	3.44 ± 2.7	3.66 ± 2.58	2.95 ± 2.06	0.085
Tubal factor	263 (50.9%)	136 (50.4%)	37 (42.0%)	0.303
Male factor	64 (12.4%)	25 (9.3%)	7 (8.0%)	0.262
DOR	51 (9.9%)	27 (10.0%)	11 (12.5%)	0.747
PCOS	38 (7.4%)^a^	31 (11.5%)^b^	16 (18.2%)^a^	0.003
Endometriosis	33 (6.4%)	10 (3.7%)	1 (1.1%)	0.056
Unexplained/Other	68 (13.2%)	41 (15.2%)	16 (18.2%)	0.404

Data expressed as mean±SD or n(%). hsCRP, high-sensitivity C-reactive protein; BMI, body mass index; FSH, follicle-stimulating hormone; E2, estradiol; LH, luteinizing hormone; AMH, anti-mullerian hormone; AFC, antral follicle count; DOR, diminished ovarian reserve; PCOS, polycystic ovary syndrome.

‘a’, ‘b’ and ‘c’ indicate that significant differences were detected between the two groups.

The IVF cycle characteristics were listed in **Table 2**. There were no statistically significant differences among the three groups in terms of the protocol of controlled ovarian hyperstimulation or gonadotropin dose administered; the same can be said for the serum estradiol, progesterone and the endometrial thickness on the day of triggering. The number of retrieved oocytes, fertilized oocytes and good quality embryos, and the oocyte maturation rate showed no significant difference among the hsCRP groups.

**Table 2:** Basic cycle characteristics of women with low, moderate and high hsCRP.

	hsCRP (mg/L)			
Characteristic	<1 (n=517)	1-3 (n=270)	>3 (n=88)	*P* value
ICSI	126 (24.4%)	65 (24.1%)	20 (22.7%)	0.946
Stimulation protocols				0.825
GnRH agonist	500 (96.7%)	259 (95.9%)	85 (96.6%)	
GnRH antagonist	17 (3.3%)	11 (4.1%)	3 (3.4%)	
Gn dosage (IU)	2639.9 ± 1344.95	2619.9 ± 1083.5	2804.53 ± 2140.66	0.530
E_2_ on hCG (pg/mL)	3074.92 ± 1771.97	3093.61 ± 1828.71	2898.94 ± 1736.54	0.659
P on hCG (ng/mL)	1.04 ± 0.58	1.03 ± 0.59	0.94 ± 0.57	0.365
Endometrial thickness (mm)	12.32 ± 2.36	12.37 ± 2.51	11.85 ± 2.29	0.195
No. of oocytes retrieved	12.09 ± 5.71	12.81 ± 6.21	12.94 ± 5.96	0.174
No. of MII oocytes	10.01 ± 4.92	10.55 ± 5.20	10.75 ± 5.19	0.218
No. of 2PN embryos	7.94 ± 4.13	8.32 ± 4.37	8.34 ± 4.37	0.415
Oocyte maturation rate	0.84 ± 0.15	0.87 ± 0.73	0.84 ± 0.15	0.543
No. of good quality embryos	7.21 ± 3.94	7.39 ± 4.06	7.47 ± 3.96	0.751
No. of frozen embryos	3.52 ± 2.79	3.63 ± 2.81	3.53 ± 2.76	0.886
Stage of embryo transfer				0.328
Cleavage embryos	357 (69.1%)	173 (64.1%)	57 (64.8%)	
Blastocyst	160 (30.9%)	97 (35.9%)	31 (35.2%)	

Data expressed as mean±SD or n(%). ICSI, intracytoplasmic sperm injection; Gn, gonadotropin; MII, mature oocytes; 2PN, fertilized oocytes with two pronuclei.

**Figure 1:**
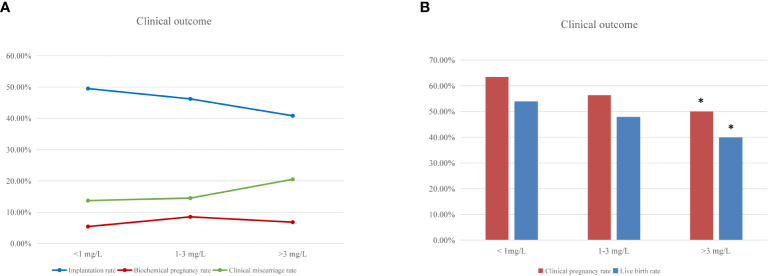
IVF clinical outcomes according to woman’s hsCRP level. **(A)** Percentage of implantation, biochemical pregnancy and clinical miscarriage rate after fresh embryo transfer cycles among women with low (<1 mg/L), medium (1-3 mg/L) and high (>3 mg/L) hsCRP level. **(B)** The frequencies of clinical pregnancy and live birth after fresh embryo transfer cycles according to the hsCRP levels. “*” means statistically significant difference (P<.0.0167) for clinical pregnancy and live birth rate in the high hsCRP group compared with low hsCRP group.

Regarding clinical outcomes, implantation rate and clinical miscarriage rate were not significantly different among the three groups, although there was a tendency to poorer results in women with high hsCRP levels (**Figure 1A** and **Table 3**). However, the clinical pregnancy rate was significantly lower in the high hsCRP group compared with that in the low hsCRP group (50.0% versus 63.4%; P<0.0167), which contributed to a significant decrease in birth rate (39.8% versus 53.8%; P<0.0167) (**Figure 1B** and **Table 3**).

**Table 3:** IVF outcomes of women with low, moderate and high hsCRP.

	hsCRP (mg/L)			
Characteristic	<1 (n=517)	1-3 (n=270)	>3 (n=88)	*P* value
Implantation rate	395 (49.5)	183 (46.2)	53 (40.8)	0.143
Biochemical pregnancy rate	28 (5.4)	23 (8.5)	6 (6.8)	0.244
Clinical pregnancy rate	328 (63.4)^a^	152 (56.3)^b^	44(50.0)^a^	0.021
Clinical miscarriage rate	45 (13.7)	22 (14.5)	9 (20.5)	0.492
Live birth rate	278 (53.8)^a^	129 (47.8)^b^	35 (39.8)^a^	0.029

Data expressed as n(%).‘a’and‘b’indicate that significant differences were detected between the two groups. Statistical significance is defined as P<0.0167.

Statistical analysis was performed using Chi-square test and Bonferroni-adjusted test.

**Figure 2:**
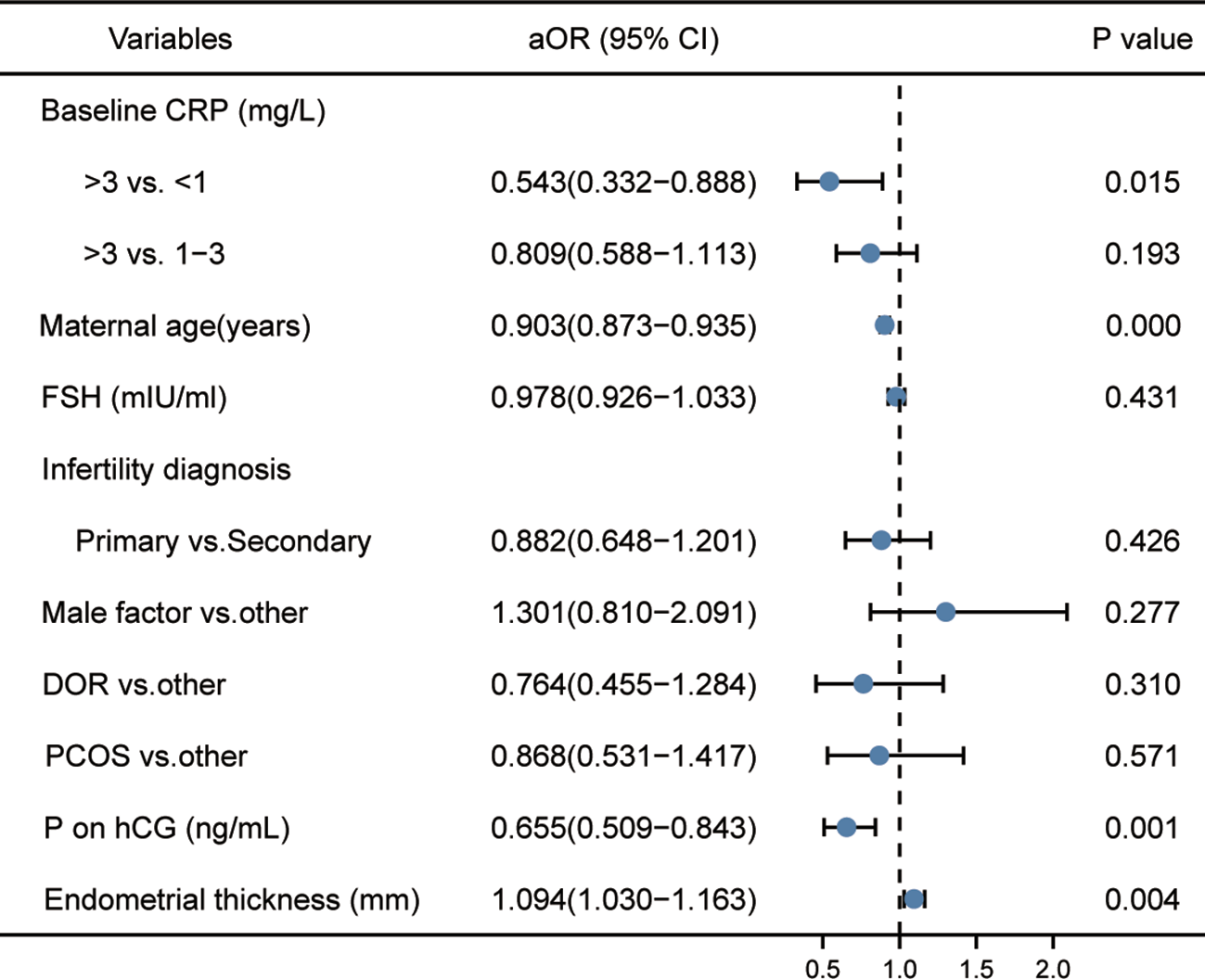
Logistic regression analysis of potential factors associated with live birth rate. ‘aOR’ indicates that the odds ratio (OR) and 95% CI were adjusted for potential confounding factors.

As shown in **Figure 2**, the logistic regression analysis showed that high hsCRP levels, compared with low hsCRP levels (aOR: 0.543 (0.332–0.888); P=0.015), was significantly associated with a reduction in live birth rate per fresh ET. Maternal age and the serum progesterone and the endometrial thickness on the day of triggering were also associated with live birth rate. However, the distribution of these factors was similar in the three hsCRP groups. No other factor was found to have an influence on the rate of live birth.

## Discussion

In the present retrospective study on women of normal weight undergoing their first fresh ET cycles for IVF treatment, reduced clinical pregnancy and live birth rates were observed in women with high baseline serum levels of hsCRP (>3 mg/L) compared with women with low levels of hsCRP (<1 mg/L). In addition, our study found that high baseline serum levels of hsCRP were associated with the reduced live birth rate, independently of several potential confounding factors, such as maternal age, baseline FSH levels, infertility diagnosis, infertility factors, and the serum progesterone and the endometrial thickness on the day of triggering.

A previous prospective study demonstrated a significant decrease in CRP concentrations prior to ovarian stimulation in women who became pregnant compared with those who did not following IVF treatment (621 ± 140 ng/mL versus 1,007 ± 131 ng/mL; P=0.047). Such a difference was shown to be more pronounced in women with diminished ovarian reserve (DOR), suggesting that higher CRP levels could lead to lower pregnancy rates and that serum CRP levels had a greater sensitivity for predicting no clinical pregnancy in women with DOR (14). In agreement with these findings, the present study observed a significantly higher rate of clinical pregnancy in the low hsCRP group than in the high hsCRP group (63.4% versus 50.0%; P<0.0167). Notably, Buyuk et al. (14) included only a very small number of participants (n=38) in their analysis, comprising 11 pregnant women and 27 non-pregnant, and had relatively lower baseline CRP levels overall. In addition, two other studies found that the baseline concentrations of CRP were lower in women who conceived compared with women who failed to conceive, although this difference was not significant; however, in the study by Sack et al., the median CRP concentrations in the serum of both groups of women were <2 mg/L, thus potentially weakening the difference between the groups (15, 22). Furthermore, two small retrospective studies indicated that there was no association between circulating CRP levels measured before ovarian stimulation and IVF success (17, 18). Previous findings have supported that female age and BMI are strongly associated with pregnancy rates in women undergoing IVF (23). As women age, the quality and quantity of their oocytes decline, leading to decreased IVF success rates (24). However, women who achieved pregnancy were at significantly younger ages than those who did not in three studies (14, 15, 22), and the female age was not provided in one study (17), which may influence the association between CRP levels and clinical pregnancy rates following IVF. Seckin et al. (18) included study subjects with high CRP levels (3.61 ± 2.86 and 3.24 ± 2.68 mg/L) and high BMI (25.9 ± 3.9 and 25.3 ± 4.0 kg/m^2^) in both the pregnant and non-pregnant groups; thus, it was difficult to detect the effects of low-grade inflammation on pregnancy outcomes.

In the present study, the sample size allowed for a detailed analysis stratified by hsCRP levels, comprising 875 cycles, with 517 women with low hsCRP levels, 270 women with medium hsCRP levels and 88 women with high hsCRP levels. The maternal age was similar among the three hsCRP groups. Additionally, obesity may result in the increased secretion of pro-inflammatory cytokines, including TNF-α, IL-6 and CRP, causing chronic inflammation, and the impaired quality and development of embryos. Adipocytes also produce adipokines, which promote the additional release of pro-inflammatory cytokines (25, 26). Furthermore, female obesity can negatively and significantly affect pregnancy outcomes following IVF, regardless of an inflammatory milieu (3, 27). Therefore, there may be differences in the association between obesity-associated and non-obesity-associated inflammation and reproduction. Recently, a prospective study found that high preconception CRP levels (≥1.95 mg/L) were associated with a significant decrease in the number of clinical pregnancies and live birth rates among women attempting to conceive naturally. However, this association was attenuated following the adjustment for BMI (12). On the whole, BMI is a confounding factor for the association between CRP levels and pregnancy rates, as previously described (13), and hsCRP has been shown to function as an indicator of women who are at a risk of impaired fertility. To our knowledge no data are available in the literature on the effects of elevated baseline levels of hsCRP on IVF outcomes in women of normal weight. Our study is therefore unique, since it was demonstrated that high serum levels of hsCRP were significantly associated with a decreased clinical pregnancy rate in women with a BMI <25 kg/m^2^ who underwent their first IVF treatment. Finally, as regards the clinical outcomes following IVF treatment, in addition to clinical pregnancy, we further compared the rate of implantation, miscarriage and live birth rate between the three hsCRP groups. This allows for a more comprehensive assessment of the association between low-grade inflammation and IVF outcomes.

The finding of the present study is consistent with that reported by Brouillet et al. (11), suggesting that high circulating levels of hsCRP during preconception could impair oocyte developmental competence, embryo quality or endometrial receptivity. Previous study has observed that the serum CRP concentrations were significantly elevated in the early phase of anovulatory cycles compared to those of ovulatory cycles (28). It has also been proposed that there is a strong association between the concentrations of CRP in serum and follicular fluid, revealing a direct association between systemic and reproductive system inflammation (29). Furthermore, in IVF, some studies have reported increased serum resistin levels, and follicular TNF-α, IL-8 and IL-12 levels are negatively associated with the number (30) and quality of oocytes (31, 32). Notably, our study found that the number of retrieved oocytes, fertilized oocyte and good quality embryos did not differ significantly among the three hsCRP groups; however, the clinical pregnancy rates were significantly decreased in women with high levels of CRP, suggesting that poor pregnancy outcomes may, in part, be attributed to abnormalities in embryo development, altered endometrial receptivity or a limited ability to predict the embryo quality by the conventional assessment of embryo morphology. For instance, Radin et al. (33) showed that high preconception hsCRP levels reduced pre or post-implantation survival of male embryos among women attempting to conceive naturally, and that daily LDA treatment prior to conception could decrease the serum hsCRP concentrations, restoring the normal offspring sex ratio. This harmful effect of higher maternal inflammation on male embryos is consistent with the results from *in vivo* animal research (34). Furthermore, the exposure of metaphase-II mouse oocytes to recombinant mouse IL-6 (50, 100 and 200 ng/ml) can result in a dose-dependent deterioration in microtubule and chromosomal alignment, suggesting that elevated IL-6 levels can affect embryos development *via* the impairment of microtubule and chromosomal structures (35). Although direct evidence is lacking, the expression of growth differentiation factor 9 (GDF9) and bone morphogenetic protein 15 (BMP15, also known as GDF9b) in other organs is influenced by inflammation (36, 37). It is reasonable to assume that inflammatory processes in the ovaries can affect the delicate interaction between oocyte and cumulus cells, thus negatively affecting both the meiotic and cytoplasmic maturation of oocytes. To date, no data have been reported regarding the association between CRP concentrations and the aneuploidy rate. Future research on the effects of serum and follicular CRP concentrations on embryo aneuploidy may be helpful in determining the role of inflammation in human embryo quality and development. Concerning the impact of chronic inflammation on endometrial receptivity, previous research has indicated that an excessive upregulation of inflammatory responses in decidual cells may reduce the window of receptivity and lead to implantation failure following IVF (38). Furthermore, the elevated expression of pro-inflammatory proteins has been found in endometrial fluid aspirated immediately prior to ET in those who did not become pregnant in comparison to those who became pregnant, revealing an increased inflammatory state in the non-implantation endometrium (5).

Some limitations of our study should be noted. First, this was a retrospective study, and thus, potential bias could not be fully avoided. Second, no definite conclusions can be drawn from the results on the mechanisms through which low-grade inflammation affects IVF outcomes. Thus, the mechanisms underlying the decreased clinical pregnancy rates in women of normal weight with high baseline serum levels of hsCRP need to be properly addressed. Finally, the limited sample size of the high hsCRP group may affect the statistical significance of the results.

## Conclusion

The present study provides preliminary evidence that, among women of normal weight undergoing their first IVF treatment, high baseline serum levels of hsCRP (>3mg/L) are associated with reduced clinical pregnancy and live birth rates following fresh ET cycles compared with low serum levels of hsCRP (<1mg/L). This indicates that low-grade inflammation may be harmful to IVF outcomes, independently of adiposity. Moreover, high serum hsCRP levels was found to be a factor affecting the live birth rate. The measurement of serum hsCRP concentrations prior to commencing ovarian stimulation may be a potentially useful test for the personalized clinical management of women undergoing IVF.

## Data availability statement

The raw data supporting the conclusions of this article will be made available by the authors, without undue reservation.

## Ethics statement

The studies involving human participants were reviewed and approved by Research Ethics Committee of the First Affiliated Hospital of Zhengzhou University. The patients/participants provided their written informed consent to participate in this study.

## Author contributions

GL and HZ conceived of and designed the study. GL, HJ and FL selected the population to be included and excluded. HZ, XL and FZ collected and analyzed clinical data. HJ and YS reviewed the data. GL and HZ drafted the manuscript. All authors contributed to the article and approved the submitted version.
